# Ultrasound assessment for extravascular lung water in patients with septic shock

**DOI:** 10.1186/cc14303

**Published:** 2015-03-16

**Authors:** P Pirompanich, A Wattanathum

**Affiliations:** 1 Thammasat University, Pathumthani, Thailand; 2 Phramongkutklao Hospital, Bangkok, Thailand

## Introduction

Extravascular lung water (EVLW) refers to fluid within the lung but outside the vascular compartment. Increment of EVLW was associated with mortality in critically ill patients. Extravascular lung water index (EVLWI) >10 ml/kg was found in patients with cardiogenic pulmonary edema and correlated with pulmonary capillary wedge pressure >20 mmHg. Measurement of EVLW needs sophisticated tools and an invasive method by transpulmonary thermodilution (TPTD) technique. In contrast, multiple B-lines by lung ultrasound (LUS) have been recently proposed to correlate with increased EVLW in patients with pulmonary edema. This study aims to compare three methods of LUS and EVLWI measured by TPTD to assess pulmonary edema in patients with septic shock.

## Methods

The authors prospectively enrolled 17 patients with septic shock who were admitted to the medical ICU, Phramongkutklao Hospital between September 2013 and June 2014. EVLWI was measured by TPTD (VolumeView Set, EV1000; Edwards Lifesciences) method. According to international evidence-based recommendations for point-of-care lung ultrasound 2012, three

## Methods

of LUS (LOGIQ e ultrasound; GE Healthcare) were compared to assess EVLW daily in each patient until no indication for invasive blood pressure monitoring [1]. Firstly, B-lines were measured in 28 lung zones. The total numbers of B-lines seen in each patient were counted as total B-line scores (TBS). Secondly, upper and lower BLUE points were anterior two-region scans each side marked by physician hands. Pulmonary edema was diagnosed if three or more B-lines were presented in all regions. Lastly, scanning eight regions, two anterior and two laterals per side, was considered abnormal if more than one scan per side had three or more B-lines.

## Results

A total of 40 comparisons were obtained. Significant positive linear correlations were found between TBS and EVLWI determined by TPTD (*r *= 0.637, *P *< 0.001). The TBS ≥39 has sensitivity of 91.7% and specificity of 75.0% to define EVLWI >10 ml/kg. There was low sensitivity (33.3% and 50.0% respectively) but high specificity (100% and 96.0% respectively) of the positive BLUE points and eight regions to define EVLWI >10 ml/kg.

## Conclusion

TBS is the best method for assessing EVLW compared with BLUE points and eight regions. These data support the benefit of LUS with summation of B-line scores of 28 rib interspaces for assessment of the increment of EVLW in septic shock patients.
